# Dataset of low global warming potential refrigerant refrigeration system for fault detection and diagnostics

**DOI:** 10.1038/s41597-021-00927-6

**Published:** 2021-05-27

**Authors:** Jian Sun, Piljae Im, Yeonjin Bae, Jeff Munk, Teja Kuruganti, Brian Fricke

**Affiliations:** 1 Electrification and Energy Infrastructures Division, Oak Ridge National Laboratory, One Bethel Valley Road, Oak Ridge, TN 37830 USA; 2 Computational Sciences and Engineering Division, Oak Ridge National Laboratory, One Bethel Valley Road, Oak Ridge, TN 37830 USA; 3 Buildings and Transportation Science Division, Oak Ridge National Laboratory, One Bethel Valley Road, Oak Ridge, TN 37830 USA

**Keywords:** Energy efficiency, Energy conservation

## Abstract

HVAC and refrigeration system fault detection and diagnostics (FDD) has attracted extensive studies for decades; however, FDD of supermarket refrigeration systems has not gained significant attention. Supermarkets consume around 50 kWh/ft^2^ of electricity annually. The biggest consumer of energy in a supermarket is its refrigeration system, which accounts for 40%–60% of its total electricity usage and is equivalent to about 2%–3% of the total energy consumed by commercial buildings in the United States. Also, the supermarket refrigeration system is one of the biggest consumers of refrigerants. Reducing refrigerant usage or using environmentally friendly alternatives can result in significant climate benefits. A challenge is the lack of publicly available data sets to benchmark the system performance and record the faulted performance. This paper identifies common faults of supermarket refrigeration systems and conducts an experimental study to collect the faulted performance data and analyze these faults. This work provides a foundation for future research on the development of FDD methods and field automated FDD implementation.

## Abstract (summary)

**Table:** 

Measurement(s)	fault detection and diagnostics • temperature • pressure • mass flow rate • power
Technology Type(s)	fault imposition • measurement device
Factor Type(s)	fault type • fault scenario
Sample Characteristic - Environment	refrigeration system

Machine-accessible metadata file describing the reported data: 10.6084/m9.figshare.14384321

## Background & Summary

As the most energy-intensive end-uses in the commercial buildings sector, supermarkets consume around 50 kWh/ft^2^ electricity annually, which is more than 2 million kWh of electricity per year for a typical supermarket store^1^. As the biggest consumer of energy in a supermarket, the refrigeration system accounts 40%–60% of total supermarket electricity usage, which is about 2%–3% of the total energy consumed by commercial buildings in the United States, or around 0.5 quadrillion Btu^2^. Also, the supermarket refrigeration system is one of the biggest consumers of refrigerants. Current supermarket refrigeration systems rely on high–global warming potential (GWP) hydrofluorocarbon refrigerants. Reducing refrigerant usage or using environmentally friendly refrigerant alternatives can result in significant climate benefits. Additionally, the supermarket refrigeration system can be adapted to handle flexible building loads and be integrated into grid response transactive control to balance the supply and demand of the electric grid^3^. Thus, even a small improvement in the operational reliability and efficiency of supermarket refrigeration systems can significantly save energy, improve food quality, protect the environment, and enhance the electric grid resilience.

Fault detection and diagnostics (FDD) techniques can be used to support supermarket refrigeration system operators in achieving these benefits. Similar to in other vapor compression system, typical faults that occur in supermarket refrigeration systems include refrigerant leakage, lubrication issues, evaporator icing or fouling, condenser fouling, control system problems, compressor inefficiency, condenser fan or motor issues, evaporator fan or motor issues, control valve malfunctioning, cabinet glass door frosting, and liquid line restrictions. Kim^4^ conducted an experimental study of four common faults found in a variable-speed vapor compression system: compressor fault, condenser fault, evaporator fault, and refrigerant leakage. According to Kim’s test results, the system parameters are insensitive to the compressor fault for a variable-speed compressor system because the compressor speed can be controlled to compensate for the faults. Tassou and Grace^5^ developed a FDD strategy using an artificial intelligence and real-time performance monitoring approach for refrigerant leakage detection and diagnosis to overcome difficulties associated with the inability to detect gradual leakage and to carefully determine a sensor’s optimum location. Yang *et al*.^6,7^ applied a Kalman filterbased method, extended Kalman filter- based methods, and an unknown input observer method for detection and isolation of four types of sensor faults: drift, offset, freeze, and hard-over. Given the limited amount of quality data available, Zhao *et al*.^8^ used a field test environment instead of a laboratory environment to evaluate some common chiller faults: reduced water flow rate fault, improper refrigerant charge fault, condenser fouling faults, and non-condensable gas in the refrigerant fault. Kocyigit *et al*.^9^ investigated several faults: compressor failure, restricted filter-dry, restricted electronic expansion valve, compressor valve leakage, undercharging, overcharging, a dirty condenser, and evaporator fan failure. They categorized the faults into two types: hard failures and soft failures. BehFar *et al*.^10^ presented two automated fault detection and diagnosis approaches, a data-driven method and a rule-based method, and found that the rule-based method is suitable for scenarios in which controlled variables are selected as the performance index, whereas the data-driven method performs better in the detection of energy consumption variation. BehFar *et al*.^11^ investigated supermarket equipment characteristics and common operating faults based on data collected through expert surveys, facility management system messages, service calls, and service records. According to their study, the most commonly occurring faults are refrigerant leakage/undercharge/overcharge, oil problems, evaporator iced-up/fouling, control system problems, condenser fouling, compressor inefficiency, condenser fan or fan motor problems, evaporator fan or fan motor problems, the interaction of HVAC and refrigeration systems, control valves problems, cabinet glass door frosting, and liquid line restriction. Among these faults, control system problems, refrigerant leakage/undercharge/overcharge, and compressor problems are the three most costly faults to repair.

However, compared with other building HVAC and refrigeration equipment and systems^12–15^, such as air-handling units, rooftop units, package air-conditioning, chillers, heat pumps, furnaces, and supermarket refrigeration systems have not attracted sufficient attention from researchers to conduct FDD studies^11^. This is especially true for low-GWP refrigerant supermarket refrigeration systems, which have some unique characteristics in terms of FDD, such as high discharge pressure, a multistage compression system, a frequent defrost cycle for low-temperature (LT) evaporators, high air infiltration on medium-temperature (MT) open display cases, and doored display cases. One of the key challenges is the lack of publicly available data sets to benchmark the system performance and record the faulted performance to support the low-GWP refrigerant supermarket refrigeration system FDD research. This paper identifies common faults of supermarket refrigeration systems and collects experimental data sets for a low-GWP refrigerant, CO_2_ in this case, supermarket refrigeration systems, which will serve as a guide for future research on low-GWP refrigerant supermarket refrigeration system FDD methods development and field implementation.

## Methods

**Fig. 1:**
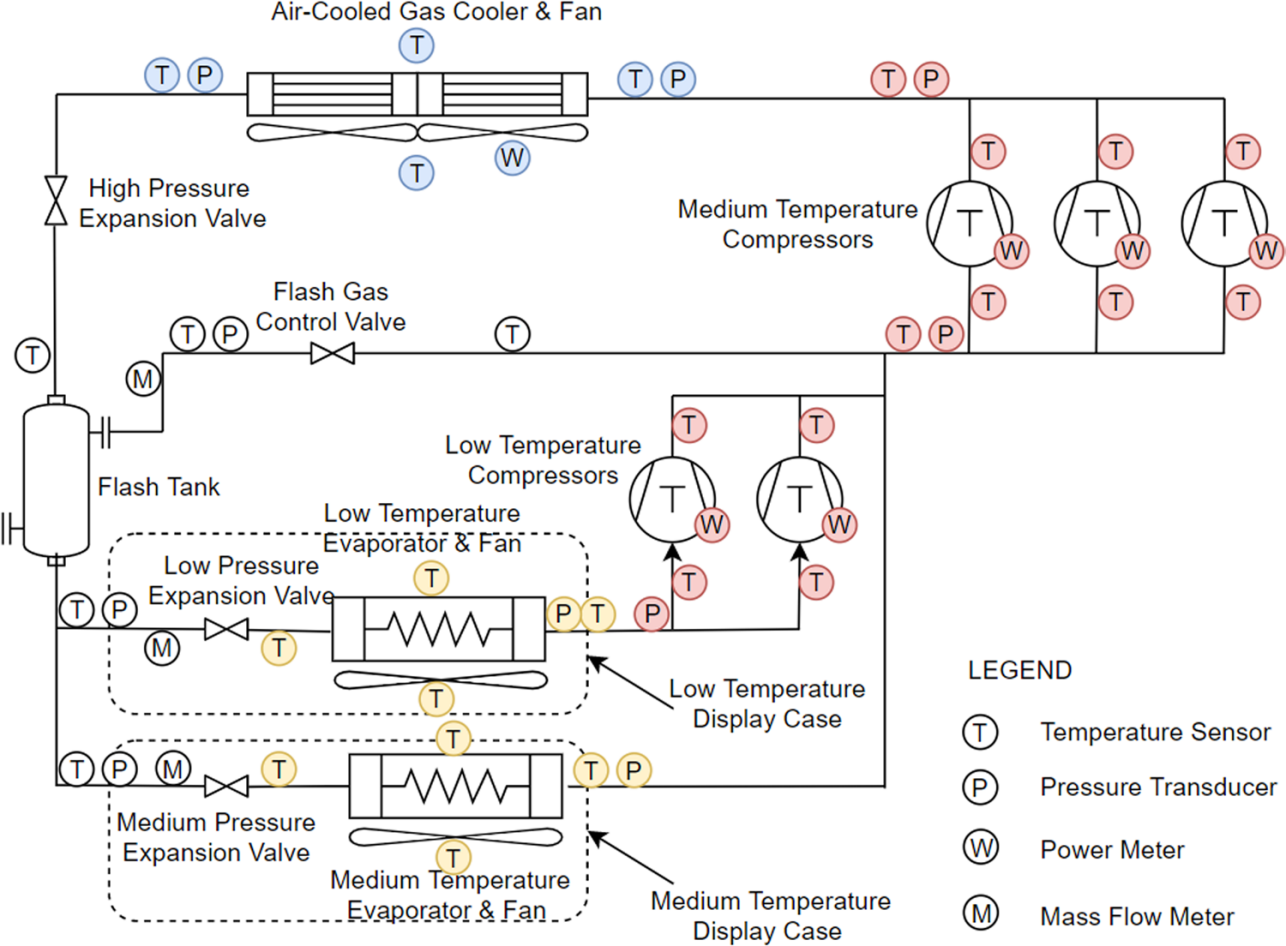
Commercial refrigeration system.

The data sets were generated through a laboratory-scale commercial refrigeration system (Fricke 2016)^16^ as shown in Fig. 1, which consists of a transcritical CO_2_ compressor rack, one MT refrigerated display case and one LT refrigerated display case, an air-cooled gas cooler, and MT and LT “false” loads.

The overall cooling capacity of this commercial refrigeration system was approximately 12.2 tons (43 kW), with a LT cooling capacity of approximately 2.6 tons (9 kW) at an evaporating temperature of −22 °F (−30 °C) and a MT cooling capacity of approximately 9.6 tons (34 kW) at an evaporating temperature of 20 °F (−6.7 °C). The LT load consisted of one 4-door display case and a LT false load provided by a plate heat exchanger, two electric heaters, and a glycol loop. The MT load consisted of one open display case and a MT false load provide by a plate heat exchanger, nine electric heaters, and a glycol loop.

### Compressor rack

The compressor rack contained two LT compressors and three MT compressors using a CO_2_ refrigerant. Each set of compressors consisted of one primary variable-speed compressor (capable of modulating capacity from 10% to 100%) and one or two secondary fixed-speed compressors. The LT compressors operated in a subcritical mode, and the MT compressors could operate in a subcritical and supercritical mode. When the refrigeration load was low, the load was handled by a primary variable-speed compressor that could modulate its capacity according to the load demand, and the secondary fixed-speed compressor was turned off. If the load exceeded the capacity of the primary variable-speed compressor, the secondary fixed-speed compressor was brought online, and the primary variable-speed compressor modulated its capacity to match the load. The target suction pressures (or suction saturation temperatures) were set based on the coldest temperature required by the display cases that those compressors served (either MT or LT). Each display case had a temperature setpoint, and the refrigerant flow to the evaporator coil of the case was modulated to maintain this temperature. Therefore, the refrigeration system power consumption could be controlled by changing these setpoints in addition to more traditional on/off means such as shutting off a compressor or compressors or shutting off refrigerant flow to one or more display cases. Each of these “control levers” resulted in different power and thermal responses of the system. Specifications for the compressors are shown in Table 1.

**Table 1:** Compressor specifications for a laboratory-scale refrigeration system.

Compressor type	Temperature level	Capacity control	Model	Refrigerating capacity, kBtu hr^−1^ (kW)*	Power, kW*
Reciprocating	LT	Fixed	2KSL-1K	19.0 (5.57)	1.34
Reciprocating	LT	Fixed	2MSL-07K	12.0 (3.52)	0.82
Reciprocating	MT	Variable	4MTC-10K	38.0 (11.1)	9.66
Reciprocating	MT	Fixed	4MTC-10K	39.0 (11.4)	9.72
Reciprocating	MT	Fixed	4MTC-7K	38.5 (11.3)	9.4

*Refrigerating capacity and power are given for the following operating conditions using R-744 (CO_2_):

LT: −22 °F (−30 °C) evaporating temperature, 20 °F (−6.7 °C) condensing temperature

MT: 20 °F (−6.7 °C) evaporating temperature, 100 °F (38 °C) condensing temperature.

### Refrigerated display cases

The LT load consisted of one 10 ft (3.0 m) long 4-door vertical display case and a false load provided by a plate heat exchanger, two electric heaters, and a glycol loop. The rated capacity of the 4-door display case was 5,700 Btu hr^−1^ (1,670 W), and the false load was approximately 20,500 Btu hr^−1^ (6,000 W). The MT load consisted of one 8 ft (2.4 m) long open vertical display case and a false load provided by a plate heat exchanger, nine electric heaters, and a glycol loop. The rated capacity of the open display case was 9,600 Btu hr^−1^ (2,810 W), and the false load was approximately 92,000 Btu hr^−1^ (27,000 W). Specifications for the display cases are shown in Table 2.

**Table 2:** Specifications for refrigerated display cases.

	LT display case	MT display case
Model number	6RZLH	O5DM-NRG
Type	4-door, vertical multi-deck	Open, vertical multi-deck
Length	10 ft (3.0 m)	8 ft (2.4 m)
Rated capacity	5,700 Btu hr^−1^ (1,670 W)	9,600 Btu hr^−1^ (2,810 W)
Fan amperage	0.93 amps	0.75 amps
Lighting amperage	0.90 amps	0.40 amps
Anti-sweat heater amperage	7.99 amps	N/A
Defrost type	Electric	Off-cycle
Defrost amperage	16.29 amps	N/A

### Air-cooled gas cooler

The Luvata (model LGV8812) air-cooled gas cooler was used to reject heat from the refrigeration system by receiving discharge refrigerant vapor from the compressor rack, condensing or cooling the refrigerant, and discharging it into a flash tank. The rated heat rejection capacity of this air-cooled gas cooler was 268,000 Btu hr^−1^ (78.5 kW) for CO_2_ at an entering temperature of 242 °F (117 °C) and an exit gas temperature of 97.5 °F (36.4 °C).

### Controls

The refrigeration system was controlled through a Danfoss AK-SC 355 system controller. The main control functions include

- Compressor control to maintain the suction pressure setpoints for the LT and MT refrigeration circuits,
- High-pressure expansion valve control to maintain optimum high-side pressure, and
- Condenser fan speed control to maintain condensing pressure.

The display cases were controlled by Danfoss AK-CC 550 A case controllers that communicated with the system controller and regulated the expansion valve position, display case air temperature, defrost operation, and lighting and fan operation.

The false loads were controlled through electronic expansion valves located in the upstream refrigerant pipe entering the false load heat exchangers. The electronic expansion valve was used to maintain a refrigerant superheat temperature of 15 K upon leaving the false load heat exchanger.

### Faults and methods of fault imposition

Based on previous refrigeration system FDD studies and industry practices, we categorized supermarket refrigeration system faults into three groups: sensor faults, mechanical and electronic device faults, and control and operational faults. Several common faults are identified as follows.

Sensor faults:

- Pressure sensor failure (compressor suction side): superheat temperature
- Temperature sensor failure (compressor discharge, evaporator exit, air supply/return)

Mechanical and electronic device faults:

- Evaporator or condenser fan motor failure
- Display case door left open by mistake
- Expansion valve failure or suction side restriction
- Refrigerant leakage or overcharge
- Non-condensable in refrigerant lines
  Control and operational faults:
- Display case overstocking or evaporator air path blockage
- Condenser air path blockage
- Evaporator coil frost accumulation or defrost heater malfunction
- Excessive infiltration to the display case

We selected six of these common faults to test in an experimental environment and study their impact on the system performance. The details are presented in the following sections.

The six faults include open LT display case door, ice accumulation on a LT evaporator coil, LT evaporator expansion valve failure, MT evaporator fan motor failure, condenser air path blockage, and MT evaporator air path blockage, as shown in Table 3.

**Table 3:** Methods of fault imposition.

Fault types	Fault scenarios	Methods of fault imposition
Open LT display case door	5%, 10%, 15% of the time	Program an automatic door open device to control the door open time
Ice accumulation on LT evaporator coil	Mild, moderate, severe	Manually set the daily defrost times to build up the ice on the evaporator coil
LT evaporator expansion valve failure	Partially closed, Fully closed	Manually adjust the evaporator expansion valve opening position
MT evaporator fan motor failure	25%, 50%, 75%	Sequentially turn off three of four MT evaporator fans
Condenser air path blockage	25%, 50%, 75%	Block the condenser coil inlet air path
MT evaporator air path blockage	25%, 50%, 75%	Block the MT evaporator coil return air path

## Data Records

The data were stored on figshare^17^, a shared platform that can be accessed publicly and used to support the energy analysis and algorithm development of supermarket refrigeration system. Table 4 summarizes the test data set, which comprises a collection of 12 comma-separated value (CSV) files.

**Table 4:** Files of the test data set.

Data file	Size (M)	File description	Sample time (s)
**Fault test 1: Open LT display case door**			
BaselineTestA.csv	95.0	Baseline test	1
Fault1_DisplayCaseDoorOpen.csv	95.4	Fault test	1
**Fault test 2: Ice accumulation on LT evaporator coil**			
BaselineTestB.csv	95.5	Baseline test	1
Fault2_IceAccumulation.csv	30.7	Fault test	3
**Fault test 3: LT evaporator expansion valve failure**			
BaselineTestC.csv	95.7	Baseline test	1
Fault3_EvapValveFailure.csv	95.2	Fault test	1
**Fault test 4: MT evaporator fan motor failure**			
BaselineTestD.csv	95.1	Baseline test	1
Fault4_MTEvapFanFailure.csv	95.0	Fault test	1
**Fault test 5: Condenser air path blockage**			
BaselineTestE.csv	95.5	Baseline test	1
Fault5_CondAPBlock.csv	95.0	Fault test	1
**Fault test 6: MT evaporator air path blockage**			
BaselineTestA.csv	95.0	Baseline test	1
Fault6_MTEvapAPBlock.csv	95.2	Fault test	1

Among these six selected faults, open LT display case door, ice accumulation, and LT evaporator expansion valve failure primarily influenced LT evaporator operation characteristics. The MT evaporator fan motor failure and air path blockage had significant effects on the MT evaporator performance. The condenser operation was mainly impacted by the condenser air path blockage. All these six faults had an effect on the LT/MT compressor operation through changing the LT/MT compressor suction and discharge refrigerant states. Therefore, several sets of monitored or calculated variables were selected to demonstrate the characteristics of LT/MT compressor, LT/MT evaporator, and condenser under faulted operation. These variables are listed as follows:

Variables representing compressor characteristics (highlighted red in Fig. 1):

- LT/MT compressor discharge temperature
- LT/MT compressor discharge pressure
- LT/MT compressor suction temperature
- LT/MT compressor suction pressure
- LT/MT compressor power

Variables representing evaporator characteristics (highlighted yellow in Fig. 1):

- LT/MT evaporator supply air temperature
- LT/MT evaporator return air temperature
- Air temperature difference across LT/MT evaporator
- LT/MT evaporator approach temperature
- LT/MT evaporator exiting superheat temperature

Variables representing condenser characteristics (highlighted blue in Fig. 1):

- Condenser inlet air temperature
- Condenser outlet air temperature
- Air temperature difference across condenser
- Condenser approach temperature
- Condenser fan power

Generally, these variables can’t be obtained through manufacturer-provided measurements, which are primarily designed for control purposes rather than for FDD. Therefore, model-based methods are widely used for development of FDD algorithms, which makes these lab test data a necessity for FDD model calibration and validation.

For each fault test, a fault-free baseline test with operating conditions similar to those of a fault test was conducted. The results of the two tests were compared to evaluate the fault performance qualitatively.

**Fig. 2:**
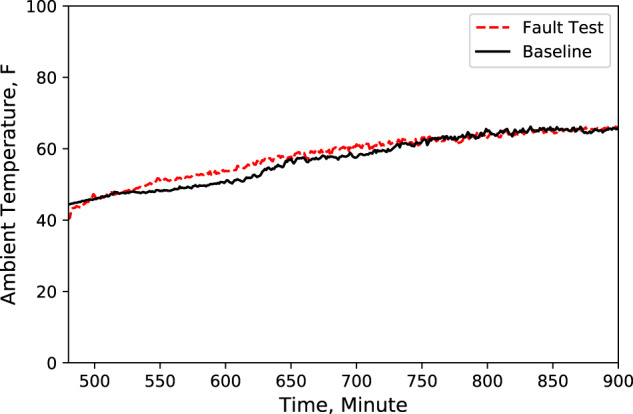
Ambient temperature. Performance comparison of a baseline test and fault test 1.

**Fig. 3:**
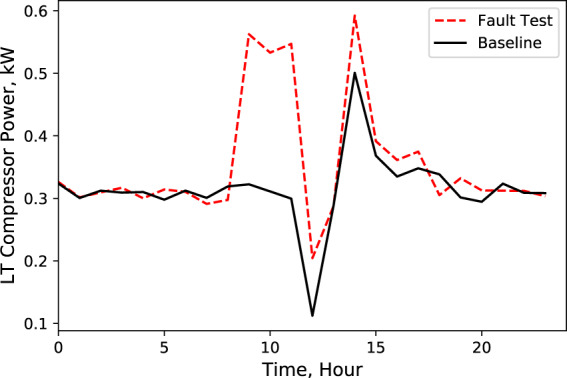
LT compressor power consumption. Performance comparison of a baseline test and fault test 1.

**Fig. 4:**
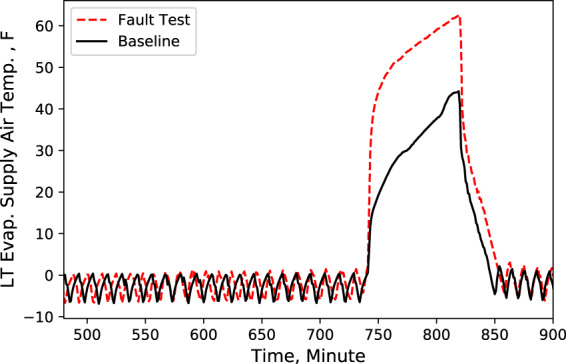
LT evaporator supply air temperature. Performance comparison of a baseline test and fault test 1.

**Fig. 5:**
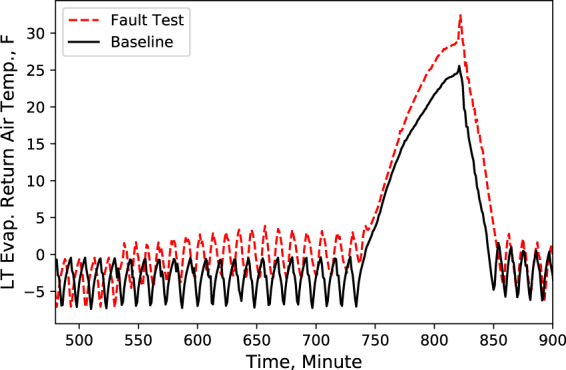
LT evaporator return air temperature. Performance comparison of a baseline test and fault test 1.

**Fig. 6:**
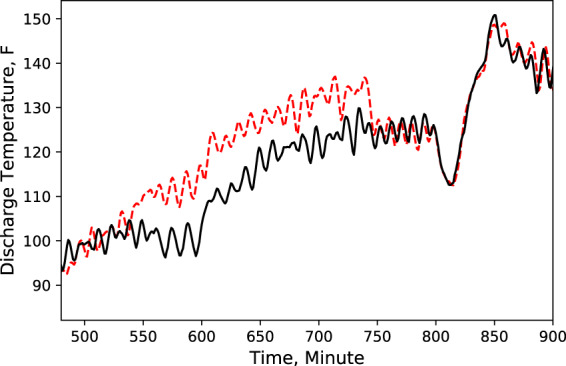
MT compressor discharge temperature. Performance comparison of a baseline test and fault test 1.

**Fig. 7:**
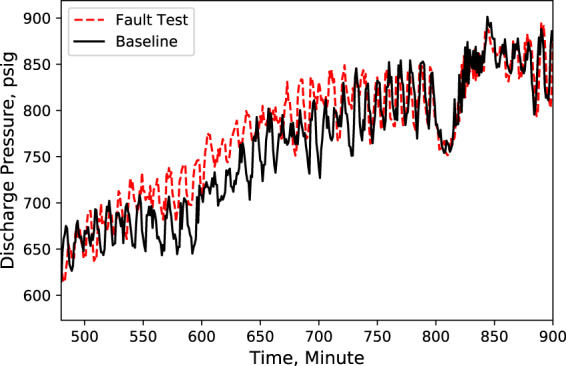
MT compressor discharge pressure. Performance comparison of a baseline test and fault test 1.

**Fig. 8:**
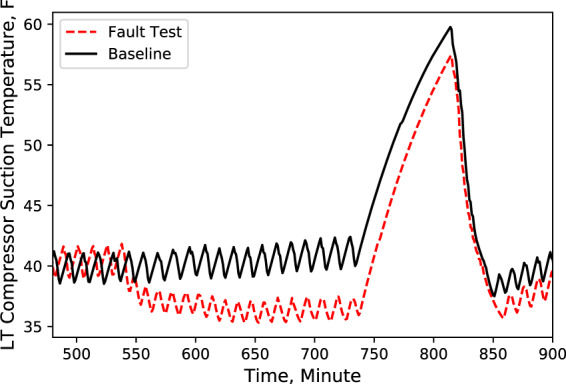
LT compressor suction temperature. Performance comparison of a baseline test and fault test 1.

**Fig. 9:**
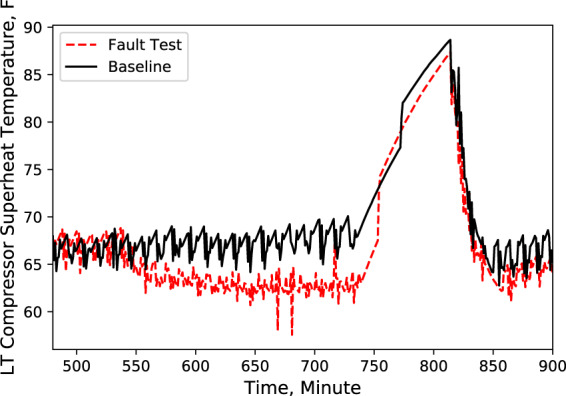
LT compressor superheat temperature. Performance comparison of a baseline test and fault test 1.

A Python code was developed to compare these key characteristics data from baseline test with data from fault test. An example of this comparison is shown in Figs. 2–9, which plots the LT display case door open faulted performance compared with the baseline performance under the test conditions: −25.6 °F (−32 °C) LT compressor suction refrigerant temperature setpoint, −6 °F (−21 °C) LT display case discharge air temperature setpoint, 16 °F (−8.9 °C) MT compressor suction refrigerant temperature setpoint, 30 °F (−1.1 °C) MT display case discharge air temperature setpoint, and 72 °F (22 °C) indoor air temperature.

According to these performance data, we noticed that LT evaporator return air temperature immediately rose (Fig. 5) after the door opened because of the warmer infiltration air from outside of the display case. However, the supply air temperature maintained the setpoint with no significant change (Fig. 4). Also, during the defrost cycle, the supply and return air temperatures rose and reached higher temperature limits than the baseline case (Figs. 4 and 5). Additionally, when the display case door opened, the MT compressor discharge temperature and pressure increased to a moderate level (Figs. 6 and 7), while Both the LT compressor suction temperature and superheat temperature drop significantly (Figs. 8 and 9). Evidently, the open door fault required more cooling load and resulted in an increase in power consumption (Fig. 3), and the compressor needed to work harder to meet the demand, which led to an increase in discharge temperature and pressure.

## Technical Validation

The commercial refrigeration system was fully instrumented to measure its performance. As shown in Fig. 1, the measurements included refrigerant temperature and pressure at the inlet and outlet of major components, such as compressors, display cases, false load heat exchangers, expansion valves, and the gas cooler, as well as refrigerant mass flow rate through the various loads, and power consumption of compressors, gas cooler fans, false load heaters, defrost heaters, and display case fans. A detailed list of the measurement points and specifications of the instrumentations is given in Table 5, and the technical quality of the data set can be understood through the accuracy of measurement.

**Table 5:** Measurement variables and instrumentation.

Measured Variable	Instruments	Range	Accuracy
**Power**			
MT 1^st^ compressor power	Watt transducer	0–80,000 W	±5% of reading
MT 2^nd^ compressor power		0–80,000 W	
MT 3^rd^ compressor power		0–80,000 W	
LT 1^st^ compressor power		0–8,000 W	
LT 2^nd^ compressor power		0–8,000 W	
**Mass Flow Rate**			
MT evaporator mass flow rate	Coriolis Mass flow meter		±5% of reading
LT evaporator mass flow rate		0–10 kg/min	
Flash tank bypass mass flow rate			
**Temperature**			
MT display case suction temperature	Thermocouple	−270- 400 °C	Greater of 1.0 °C or 0.75% for 0 to 350 °C)
LT display case suction temperature			
Flash tank vapor outlet temperature			
Gas cooler inlet/outlet temperature			
MT compressor rack inlet/outlet temperature			
LT compressor rack inlet/outlet temperature			
MT 1^st^ compressor suc./dis. temperature			
MT 2^nd^ compressor suc./dis. temperature			
MT 3^rd^ compressor suc./dis. temperature			
LT 1^st^ compressor suc. /dis. temperature			
LT 2^nd^ compressor suc./dis. temperature			
**Pressure**			
MT display case suction pressure	Pressure transducer	0–14 Mpa	±0.25% full scale
LT display case suction pressure		0–7 Mpa	
Flash tank vapor outlet pressure		0–14 Mpa	
Gas cooler inlet/outlet pressure		0–14 Mpa	
MT compressor rack inlet/outlet pressure		0–14 Mpa	
LT compressor rack inlet pressure		0–7 Mpa	
LT compressor rack outlet pressure		0–14 Mpa	

Beside the measurement, simulation models^18^ were also used to present the technical quality of the data set. A high-fidelity rigorous simulation model was created and validated with the experimental data of this low-GWP refrigerant supermarket refrigeration. Several key system operational indices, including compressor discharge temperature, gas cooler outlet temperature, LT refrigeration circuit mass flow rate, and MT refrigeration circuit mass flow rate, were compared with experimental results. Energy performance indices, such as total compressor power consumption, LT evaporator capacity, MT evaporator capacity, and air-cooled gas cooler capacity, were used to compare the simulated performance with the actual measurements. As shown in Table 6, the differences between the measured total compressor power consumption and the simulated total compressor power were within ±3.1%.

**Table 6:** Example of energy performance indices used to validate a simulation model with experimental data.

Energy performance indices		Data 1	Data 2	Data 3	Data 4
Total compressor power	Value (W)	8,854	7,469	7,003	9,246
	Difference (%)	−2.1%	3.1%	−0.4%	−0.6%

## Data Availability

A Python code was developed to process the data set to compare the baseline test results with the faulted test results. The code was stored on figshare^17^ and on a shared platform that can be accessed publicly. The data acquisition system used LabVIEW. The data file format was automatically transferred from data loggers to storage on an Oak Ridge National Laboratory local PC with sample time of 1 or 3 s.

## References

[1] US EPA. *Energy Star Building Upgrade Manual*, http://www.energystar.gov/sites/default/files/buildings/tools/EPA_BUM_CH11_Supermarkets.pdf (2008).

[2] Navigant Consulting. *Energy Savings Potential and R&D Opportunities for Commercial Refrigeration*, (2009).

[3] Booten, C., *et al*. *Development and Evaluation of Distributed Energy Resource Device Models: Electric Vehicles, Electric Water Heaters, and Commercial Refrigeration Systems*. US Department of Energy, 10.2172/1660063, (2020)

[4] Kim M, Kim MS (2005). Performance investigation of a variable speed vapor compression system for fault detection and diagnosis. International Journal of Refrigeration.

[5] Tassou SA, Grace IN (2005). Fault diagnosis and refrigerant leak detection in vapour compression refrigeration system. International Journal of Refrigeration.

[6] Yang Z, Rasmussen KB, Kieu AT, Zamanabadi RI (2011). Fault Detection and Isolation for a Supermarket Refrigeration System – Part One: Kalman-Filter-Based Methods. IFAC Proceedings Volumes.

[7] Yang Z, Rasmussen KB, Kieu AT, Zamanabadi RI (2011). Fault Detection and Isolation for a Supermarket Refrigeration System – Part Two: Unknown-Input-Observer Method and Its Extension. IFAC Proceedings Volumes.

[8] Zhao X, Yang M, Li H (2014). Field implementation and evaluation of a decoupling-based fault detection and diagnostic method for chillers. Energy and Buildings.

[9] Kocyigit N (2014). Fault diagnosis of a vapor compression refrigeration system with hermetic reciprocating compressor based on p-h diagram. International Journal of Refrigeration.

[10] BehFar A, Yuill D, Yu Y (2017). Automated fault detection and diagnosis methods for supermarket equipment (RP-1615). Science and Technology for the Built Environment.

[11] BehFar A, Yuill D, Yu Y (2018). Supermarket system characteristics and operating faults (RP-1615). Science and Technology for the Built Environment.

[12] Yu Y, Woradechjumroen D, Yu D (2014). A review of fault detection and diagnosis methodologies on air-handling units. Energy and Buildings.

[13] Katipamula, S. & Brambley, M. R. Methods for Fault Detection, Diagnostics, and Prognostics for Building Systems—A Review, Part I, *HVAC&R Research* **11**, 3–25 (2005).

[14] Katipamula S, Brambley MR (2005). Methods for Fault Detection. Diagnostics, and Prognostics for Building Systems—A Review, Part II. HVAC&R Research.

[15] Kim W, Katipamula S (2018). A review of fault detection and diagnostics methods for building systems. Science and Technology for the Built Environment.

[16] Fricke, B.A. & Sharma, V. High Efficiency, Low Emission Refrigeration System. United States, Web. 10.2172/1311267 (2016).

[17] Sun J (2021). Figshare.

[18] Sun, J., Kuruganti,T., Munk J., Dong J. & Cui B. Low global warming potential (GWP) refrigerant supermarket refrigeration system modeling and its application. International Journal of Refrigeration **126** (2021).

